# Case report of granular acute lymphoblastic leukemia and review of the literature

**DOI:** 10.1002/ccr3.1866

**Published:** 2018-11-21

**Authors:** Sangyang Jia, James Jae, Cyrus C. Hsia

**Affiliations:** ^1^ Schulich School of Medicine and Dentistry Western University London Ontario Canada; ^2^ Department of Medicine, Division of Hematology London Health Sciences Centre London Ontario Canada

**Keywords:** acute lymphoblastic leukemia, cytoplasmic granules

## Abstract

Granular acute lymphoblastic leukemia (ALL) is a rare variant of the disease that is associated with a lower remission rate to standard induction chemotherapy. Flow immunophenotyping, cytogenetics, and molecular diagnostics should be utilized to confirm the diagnosis of ALL versus acute myeloid leukemia (AML) in order to provide appropriate management.

## 1 INTRODUCTION

Diagnostic confusion can arise when acute lymphoblastic leukemia (ALL) patients present with intracytoplasmic granules which typically are found in acute myeloid leukemia (AML). Granular ALL is extremely rare in adults, and the natural history of this condition in adults is not well described. We describe a case of granular ALL in a 54‐year‐old woman and review its clinical significance in adults reported in the literature.

The presence of azurophilic granules in the cytoplasm is one of the key distinguishing features of acute myeloid leukemia (AML) which differentiates it from acute lymphoblastic leukemia (ALL). However, on rare occasions, these intracytoplasmic inclusions may be found in ALL blasts, termed granular ALL, that defy conventional teaching and diagnostic pathways.^1^ Although uncommon, granular ALL may be found at a higher incidence in pediatric patients and may be associated with a worse prognosis in this cohort.^2^ We review the case history and pathological findings in an adult patient with granular ALL and discuss the impact of granules on clinical outcomes in these ALL patients through a review of the literature.

## 2 CASE HISTORY

A 54‐year‐old woman presented with light chain multiple myeloma three years prior and was treated with VAD (Vincristine, Adriamycin, and Dexamethasone) followed by an autologous stem cell transplant resulting in a complete response. This was followed by thalidomide maintenance therapy complicated by mild peripheral neuropathy.

**Figure 1:**
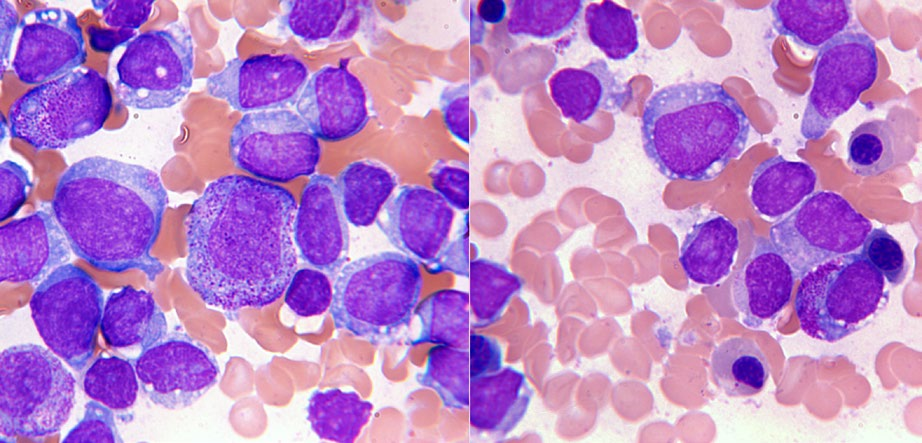
Bone marrow aspirate slides revealing some blasts with numerous cytoplasmic granules and some blasts with vacuoles

The patient subsequently developed progressive neutropenia over the course of three years. Work‐up demonstrated abnormal appearing blasts and nucleated red cells on her peripheral blood white cell differential and smears. 33% of peripheral leukocytes were described as blasts. She was admitted to hospital for further work‐up and management. A bone marrow aspirate was performed. Morphologic assessment revealed approximately 15% of the marrow blasts had numerous cytoplasmic granules suggestive of an underlying acute myeloid leukemia (Figure 1). However, flow cytometric immunophenotyping was positive for CD34, dim CD19, HLA‐DR, and cytoplasmic CD79a. CD13, CD33, cytoplasmic CD3, CD10, and MPO were negative. The findings were consistent with a precursor B‐cell acute lymphoblastic leukemia. There was no central nervous system (CNS) involvement as her cerebral spinal fluid (CSF) was negative.

Induction chemotherapy with the standard Dana‐Farber ALL protocol was instituted with vincristine, doxorubicin, prednisone and asparaginase, as well as intrathecal methotrexate, cytarabine, and hydrocortisone. She achieved a complete remission and underwent the prophylactic CNS phase of treatment consisting of intrathecal hydrocortisone, methotrexate, and cytarabine via Ommaya reservoir, as well as CNS radiotherapy, followed by the intensification phase of the Dana‐Farber protocol for eight cycles. Following the eighth cycle of the intensification phase, she developed febrile neutropenia, decreased level of consciousness, and respiratory failure requiring intubation. She succumbed to an underlying sepsis and died approximately nine months after the initial diagnosis of her ALL.

## 3 DISCUSSION

Cytoplasmic granules are one of the key morphologic features differentiating acute myeloid leukemia (AML) from acute lymphoblastic leukemia (ALL). These granules in AML are fused lysosomes and contain lysosomal enzymes and crystalline inclusions, but are rarely present in adult ALL cells.^3^ The morphology of these granules has been characterized as membrane bound vesicles and tubular arrays.^4^ In pediatric cases of ALL, granules present at a higher frequency. Cerezo et al^2^ identified 56 cases of granular ALL out of 1252 pediatric ALL patients (4.5%). These cases were Sudan black and myeloperoxidase negative and positive for the French‐American‐British (FAB) morphologic criteria. Granular acute lymphoblastic leukemia is defined by more than 5% marrow blasts having at least three azurophilic cytoplasmic granules. The Pediatric Oncology Group study also found that granular ALL was more frequent amongst FAB L2 compared to FAB L1 and that those with granular lymphoblasts had a significantly lower complete remission rate and event‐free survival.

However, it is currently unknown whether granular ALL presents a different clinical outcome in adult patients. We performed an exhaustive literature search of all cases of adult granular acute lymphoblastic leukemia and found 18 cases in addition to our case (Table 1). The mean age of the 20 adult patients was 43 years (range 20‐63) with 7 men and 12 women. The mean hemoglobin, white blood cell count, and platelet count at time of diagnosis were 7.8 g/dL, 37.3 × 10^9 ^cells/L, and 99.1 × 10^9 ^cells/L, respectively. The mean percentage of blast cells with granules was 39% (range 15‐98). All patients were MPO negative where data were available. Most patients were also CD10 positive, with two exceptions, one of which is our case. All patients where data were available were FAB group L2, which is consistent with the pediatric finding of increased granular ALL rates in that group. Most cases of granular ALL were of B‐cell lineage origin and most cases were primary leukemias. A variety of chemotherapeutic regimens were applied. The complete remission rate to induction chemotherapy was 53% which is lower than the rates reported in the literature for non‐granular adult ALL (80%‐90%).^5^ The mean overall survival at the date of publication was 8.25 months compared to a five‐year overall survival of 30% for non‐granular patients.^6^

**Table 1:** Characteristics and outcomes of 20 adult granular acute lymphoblastic leukemia cases

Author	Year reported	Age	Sex	Hgb g/dL	WBC 10^9^ cells/L	Plt 10^9^ cells/L	% peripheral blasts granular	FAB	MPO	CD10	CD19	CD33	T‐cell antigens	B cell vs T cell	Primary vs. secondary	Treatment	Complete response	Survival (mo)	Cause of death
Present case	2017	54	F	11.1	1.6	173	15		Neg	Neg	Pos	Neg			Secondary	Dana‐Farber protocol	Y	9	Sepsis
Xu^7^	2017	63	F						Neg	Pos	Pos		Neg	B	Primary				
Kishore^8^	2016	40	F	9.7	60	180	32		Neg	Pos	Pos		Neg	B	Primary		Y		
Agarwal^9^	2010	50	M	3.6	17.8	23	22		Neg	Pos	Pos	Pos	Neg	B	Primary	MCP‐841	N		Died during induction
Anand^10^	2008	30	F	5.4	20	20	22	L2	Neg	Pos	Pos	Neg	Neg	B	Primary				
Pitman^11^	2007	45	F		1.2		25	L2	Neg	Neg			Neg	B	Secondary	VAD, thalidomide and dexamethasone, hyper‐CVAD	Y		
Fulcher^3^	2006	56	F	4.1	2.9	26	10		Neg	Pos	Pos		Neg	B	Primary	Hyper‐CVAD	Y	7	
Morita^12^	2002	58	F	5.3	33.5	72	84	L2		Pos	Pos	Pos		B	Primary		Y	6	
Bolado‐Martinez^13^	1997	52	M		167		98	L2		Pos	Pos	Neg		B	Primary	TOTAL XI	N	0.5	Hemopytysis
Bolado‐Martinez^13^	1997	20	F		36		86	L2		Pos	Pos	Neg		B	Primary		N	0.75	Severe septicemia
Schwarzinger^14^	1993	38	M	8.6	18.7	113	45	L2	Neg	Pos	Pos	Neg	Neg	B	Primary	BMFT protocol	Y	8	
Tauchi^15^	1991	58	F		26.6		30	L2	Neg	Pos	Pos	Neg		B	Primary	Combination chemotherapy	Y		
Canta‐Rajnoldi^4^	1989	22	M	8.5	31.2	99	25	L2	Neg	Pos	Neg	Neg		B	Primary	Vincristine, daunorubicin, Ara‐C, prednisone	Y	27	
Canta‐Rajnoldi^4^	1989	58	M	8.2	1.7	2.3	47	L2	Neg	Pos	Pos	Neg		B	Primary	Vincristine, daunorubicin, Ara‐C, prednisone	N	8	
Canta‐Rajnoldi^4^	1989	38	M	6.3	2.2	44	41	L2	Neg	Pos	Pos	Neg		B	Primary	Vincristine, daunorubicin, Ara‐C, prednisone	N	14	
Canta‐Rajnoldi^4^	1989	21	F	9.8	80.0	243	30	L2	Neg	Pos	Neg	Neg		B	Primary	Vincristine, daunorubicin, Ara‐C, prednisone	N	7	
Hay^1^	1987	20	M	6.5	12.6	68	30	L2	Neg	Pos	Neg		Neg	B	Primary	Vincristine, daunorubicin, asparginase, prednisolone	N	7	
Hay^1^	1987	54	F	7.1	0.4	60	25	L2	Neg	Pos	Pos		Neg	B	Primary	Vincristine, daunorubicin, asparginase, prednisolone	N	1	Aspergillus pneumonia
Fradera^16^	1986	45	F	14.7	158	264	35						Pos	T	Primary	Vincristine, Prednisone	Y	12	Sepsis

Hgb, hemoglobin; WBC, white blood cell; Plt, platelet; FAB, French‐American‐British Classification; MPO, myeloperoxidase; VAD, vincristine, doxorubicin, dexamethasone; CVAD, cyclophosphamide, vincristine, doxorubicin, dexamethasone.

The heterogeneity in presentation of these cases imparts difficulty in the analysis and categorization of adult granular acute lymphoblastic leukemia. Limitations of our review of the literature include missing data in the described cases as well as the diversity in patient, disease characteristics, and treatment modalities described. The rarity of granular ALL also limits statistical analysis, further generalization, and adequate assessment of subgroups of ALL.

## 4 CONCLUSION

An accurate diagnosis is critically important for treatment implementation and prognosis in acute leukemia. Diagnostic confusion may arise in acute lymphoblastic leukemia with cytoplasmic granular inclusions resembling that of acute myeloid leukemia. It is important to confirm the type of acute leukemia with flow cytometric immunophenotyping in all cases. In this review of the literature, granular ALL in adults results in a worse prognosis compared to non‐granular adult ALL patients which is consistent with the finding in pediatric granular ALL patients.

## CONFLICT OF INTEREST

None declared.

## AUTHOR CONTRIBUTION

SJ and JJ: wrote the initial manuscript. CCH: provided case and revised the manuscript.
